# Bioactivity properties of hydroxyapatite/clay nanocomposites

**DOI:** 10.1038/s41598-023-45646-7

**Published:** 2023-11-14

**Authors:** Edwin Andrew Ofudje, James Asamu Akande, Ezekiel Folorunso Sodiya, Gabriel O. Ajayi, Adeniyi John Ademoyegun, Abdullah G. Al-Sehemi, Yasar N. Kavil, Ammar M. Bakheet

**Affiliations:** 1 Department of Chemical Sciences, Mountain Top University, Prayer City, Ogun State Nigeria; 2 Department of Chemistry and Biochemistry, Caleb University, Imota, Lagos State Nigeria; 3 Department of Biochemistry, Mountain Top University, Prayer City, Ogun State Nigeria; 4 Department of Chemical Sciences, Adekunle Ajasin University, Akungba, Ekiti State Nigeria; 5 Research Center for Advanced Materials Science (RCAMS), King Khalid University, 61413 Abha, Saudi Arabia; 6 Marine Chemistry Department, Faculty of Marine Sciences, King Abdulaziz University, P.O. Box 80207, 21589 Jeddah, Saudi Arabia; 7 ChemEconomy, Non Profit Organization for Environment Protection, 46429 Yanbu, Saudi Arabia

**Keywords:** Chemistry, Materials science, Nanoscience and technology

## Abstract

The need for bioactive and non-toxic biomaterials is on a high demand in tissue engineering applications nowadays. Hydroxyapatite (HAp) is the chief constituent of teeth and bones in mammas. One of the major challenges with the use of HAp in engineering application is its brittleness and to overcome this, it’s important to react it with a material that can enhanced it’s fragility. To this end, HAp and HAp/clay nanocomposites were developed via wet chemical process to mimic natural HAp and to equally confer special properties such as mechanical properties, high surface area, crystallinity, high porosity, and biocompatibility on the biomaterial. The functional groups properties of the as-prepared nanocomposites analyzed by FT-IR showed that the HAp and clay posed reactive centers such as Al–Al–OH, Si–Si–OH, Si–O, PO_4_^3−^, –OH, and Si–O–Al. The XRD results confirmed the formation of HAp/clay nanocomposite, while SEM and TEM images showed the morphologies of the prepared nanocomposites to be round shape particles. Besides, EDX result revealed the Ca/P ratio of HAp and HAp-C to be lower than that of stoichiometric ratio (1.67) which implies the presence of K, Na, Ca, Mg, Si and Al in the HAp/clay nanocomposite. The mechanical properties of the apatite were greatly enhanced by the addition of clay. The physiological behaviour of the fabricated apatite composites in saline solution showed steady increase in the values of the saline pH of the various biomolecules until day 5 and became fairly constant at day 7 with pH range of 7.30–7.38. Though the saline solution was acidic at the beginning due to dissolved carbon dioxide, the pH of the saline solution containing the nanocomposites gradually became neutral and fairly alkaline over time as a result of the presence of Lewis basis structures in the composites which helps in neutralizing the acidic solution. Furthermore, proliferation of apatites particles onto the surface of the nanocomposites was observed after treatment with simulated body fluids (SBF) media for 7 days. Thus, HAp/clay nanocomposites can be useful biomaterials in bone tissue engineering.

## Introduction

Hydroxyapatite (HAp) is one of the most sought after biomaterial due to its versatile role in bone implantation application owing to its likeness with natural bone material^1,2^. It’s the most commonly known apatitic among the calcium phosphate ceramic family. Hydroxyapatite Ca_10_(PO_4_)_6_(OH)_2_, is the main constituent of hard tissues which is inexpensive material with vast range of medical and industrial applications^1–3^. Although, polymer materials have great significant role in the preparations of artificial organs, their engineering applications are restricted because they are neither biocompatible nor bioactive^1^. On the other hand, various studies have revealed that HAp biomaterial showed no toxicity, pyrogenetic response and inflammatory response during tissue engineering applications^2^. The biomaterial demonstrates tremendous fibrous tissue formation during bone or teeth implant and can attach directly to the host bone^2^. In spite of the proven capability of HAp to enhance bone attachment, its span life term feat is often limited due to challenges such as surface adhesion, speedy dissolution following loss of bone–bonding, structural weariness due to lack of good mechanical property, and the formation of particulate debris^3^. Thus, surface coating of hydroxylapatite promotes direct physiochemical attachment with bone and facilitates quick implant fixation and the growth of a stable bone interface within the biomaterial structure^4^.

Metallic biomaterials are widely used as surface modification for HAp in medical treatments with several metals that are able to be produced biocompatibility and intimate contact with living tissues. However, due to the released of contaminants into the human body after prolong usage often limits the applications of metallic biomaterials during implant process which is a major setback^5–9^. Furthermore, since these metallic biomaterials are surrounded by body fluids, they are prone to corrosion degradation^5–9^. The introduction of steel such as Ti or Co–Cr alloys for long term in the body has been reported to be linked with high risk of systemic hypersensitivity reactions and development of cutaneous, however, with high amount of these metals compared to natural bone tissue could results in stress shielding and subsequent osteopenia^10^. Bartl^11^ reported that in load-bearing implants, septic or aseptic relaxing of the implant may impede the transport of the bidding force to the dental implant and the bone, resulting in mechanical malfunction when the weariness potency of the dental implant is attained. Shearier et al*.*^12^ observed that low cytocompatibility of pre-osteoblasts and endothelial cells occur as a result of the excessive released of Zn ion from pure Zn during in vitro cytotoxicity study. Though Mg implant shows relatively nontoxic degradation products, literature report has it that cell behaviors and functions through high pH could be affected due to speedy degradation of Mg during implants^13^.

In an attempt to overcome these challenges, surface modification with bioactive ceramics such as clay to form nanocomposite is seen as the surest way to improve bone-biding ability of the implant material and also improve the mechanical vigor (load bearing ability) of the biomaterial which will subsequently increase its life span without any implication. The application of clay in the production of nanoparticle is very demand. Natural clay particles have hydrophilic property, specific surface area, swelling capacity, nontoxicity, high adsorption capacity, and high solubility^14,15^. Biomaterials coated with clay minerals have been showed to bond strongly to the silicate layer which will not aggregate and non-toxic to the host cells^16–18^. Ambre et al.^19^ used organically modified clay mineral to prepared mineralized hydroxyapatite nanocomposite which demonstrated great medical applications.

In this study, we present the use of natural clay as a bioactive ceramic for the formation of scaffold hybrid composite with hydroxyapatite for tissue engineering application in order to resolved HAp challenges among which includes speedy dissolution during implant, structural weariness due to lack of good mechanical property and to also avoid the toxicity normally encounter due to the use of metallic biomaterials. The microstructural elucidation was evaluated using XRD, EDX, FT-IR, SEM and TEM analytical techniques, while the bioactivity property of HAp/clay was equally performed and so documented. The bioactivity study of the prepared composite was observed by the immersion of the as-synthesized biomaterial nanocomposite inside simulated body fluid (SBF).

## Experimental

### Synthesis of HAp

HAp was prepared by stirring 0.1 M Ca(NO_3_)_2_·6H_2_O solution in 250 mL volumetric flask for 20 min using magnetic stirrer. Thereafter, 0.06 M (NH_4_)H_2_PO_4_ was introduced and the solution pH was kept at pH 11 by adding ammonia solution. The mixture was allowed to age for 24 h, filtered through centrifugation for 10 min at 4500 rpm and cleansed with deionized water. The semi-liquid formed was placed in an oven overnight at 100 °C.

### Synthesis of HAp-clay composite

Dried HAp sample was crushed using a mortar and pestle and sieved using a mesh with a nominal opening of 200 μm. In other to prepare the hydroxyapatite-clay (HAp-C) composite, different proportions of HAp and clay samples were mixed together and sintered. Three different types of HAp-C were produced, by varying the proportion of clay to HAp. The HAp was mixed with clay in different ratios by volume (70:30, 60:30 and 50:50). Prior to the mixing, the natural clay sample was thoroughly washed and dried at a temperature of 100 °C. Water was used as the binding agent and it was added until the mixture of HAp and clay turned paste. The resulting mixtures were sintered in a furnace at a temperature of 1000 °C at a heating rate of 3 °C/min for 3 h. The resulting HAp-C sample was turned into powdery form using pestle and mortar to obtained fine particle size. The HAp-C fabricated was processed further to form scaffold by combining it with a pore forming agent of ammonium bicarbonate (AB) and the samples were compressed by hydraulic press to form pellets using a compaction pressure of 50 MPa. Thereafter, these pellets were heated at a temperature of 1000 °C inside a box furnace to yield interconnected pores of HAp-C-AB scaffold. The porosity, densification of the scaffold produced were determine using the method described by Ofudje et al. ^20^, while SEM was used to investigate the surface morphology of the as-synthesized scaffold.

### Characterization of HAp-C

The elemental composition of raw materials and HAp-C composites were done with Dispersive X-ray Spectroscopy (EDX), the crystalline determination was done using X-ray Diffraction in which diffraction peaks were collected at 2θ = 10°–60° in an incremental step of 0.02 with CuKα (γ = 1.54178 Å) radiation. The morphologies of the nanocomposites were studied using TEM; Tecnai 20 G2 FEI, Netherland Scanning Transmission Electron Microscope. Samples of HAp-C particle were dissolved in ultrapure water and sonicated for 10 min. Thereafter, the particle dropped on copper grid and kept under infrared lamp prior to measurement. FT-IR spectroscopy was used to investigate the different functional groups available on the surface of the biomaterial by using Bruker Optics, TENSOR 27 series FT-IR spectrometer. Samples ratio of 1:99% of HAp-C powder and KBr were made by mixing followed by compression using hydraulic press at a compaction pressure of 50 MPa to produce pellet.

### Biocompatibility assay

**Figure 1:**
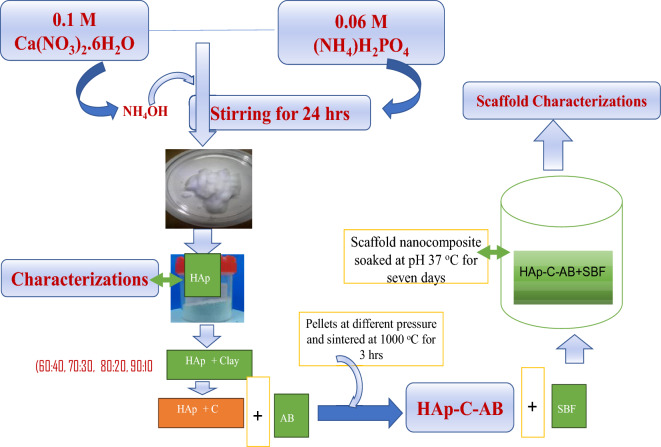
Flow chart for the production of HAp-C-AB composite and its biological applications.

For the bioactivity study, the pellets made were placed in an abrade bottle containing 150 mL of SBF media which was earlier prepared following the method of Kokubo and Takadama^21^. The abrade bottle containing the mixtures of SBF and pellets were kept in water bath shaker maintained at 37 °C for soaking. After 1 week of soaking, the bottles were recovered, samples removed, rained in distilled water followed by drying in an oven. The expected interactions on the pellets surface were cross-examined by FT-IR, XRD and SEM analyses. The solution pH was also monitored in order to observe the dissolution of the scaffold in SBF solution. Figure 1 shows the flow chart for the production of HAp-C-AB and the bioactivity study.

## Results and discussions

### Characterizations

**Figure 2:**
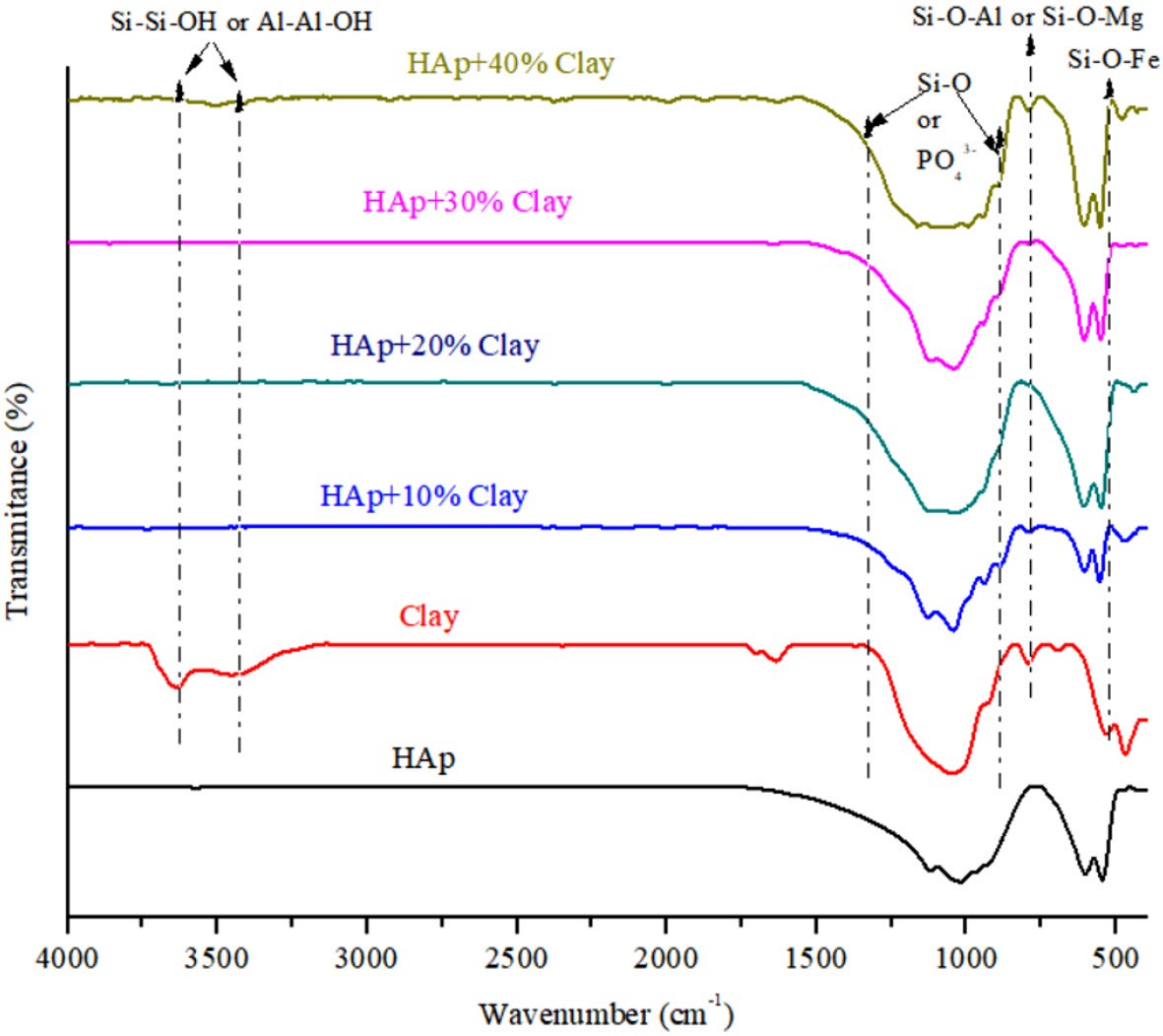
FT-RT spectra of natural clay, synthesized HAp and HAp/clay nanocomposites.

The characteristic bands matching various functional groups in the prepared pure HAp, clay and the various composites formed are shown in the FT-IR spectrum represented in Fig. 2. The obtained characteristic bands of kaolinite and montmorillonite were observed in the natural clay at 3615, 3620, 1102, 904, 745, 534, and 462 cm^−1^ which were attributed to Si–Si–OH, or Al–Al–OH, Si–O, Si–O–Al and Si–O–Mg, Si–O–Fe respectively^22,23^. This inferred that the clay sample contain mixed layer charge of trivalent Al^3+^, Fe^3+^ or bivalent Mg^2+^ ion which could play vital roles in biomedical activity. The HAp FT-IR analysis reveals that the small peak observed at 3545 cm^−1^ is ascribed to O–H vibration of water molecules which has been eliminated by heat treatment, while the phosphate peaks were observed at 1120 cm^−1^ and 570 cm^−1^. The presence of carbonated apatite was noticed with the peaks appearing at 1430 cm^−1^ and 887 cm^−1^. The FT-IR spectra of HAp/clay composites show functional groups of HAp and clay respectively. In the composites are vibration’s structure corresponding to Si–Si–OH, Al–Al–OH or OH from adsorbed water observed at 3620, 3635, 3545 cm^−1^. Bands belonging to Si–O, Si–O–Al and PO_4_^3−^ were seen at 1105, 906,1125 cm^−1^ and it was observed to be broader which could be due to the incorporation of clay particles into the structure of the apatite. This observation confirmed the formation of HAp-clay composite. Furthermore, Si–O stretching at 780–650 cm^−1^, Al-O vibration at 605 cm^−1^, PO_4_^3−^ stretching at 577 cm^−1^ and Si–O-Fe deformation noticed at 495 cm^−1^ were other prominent peaks present on the surface of the HAp/clay composite^24,25^.

**Figure 3:**
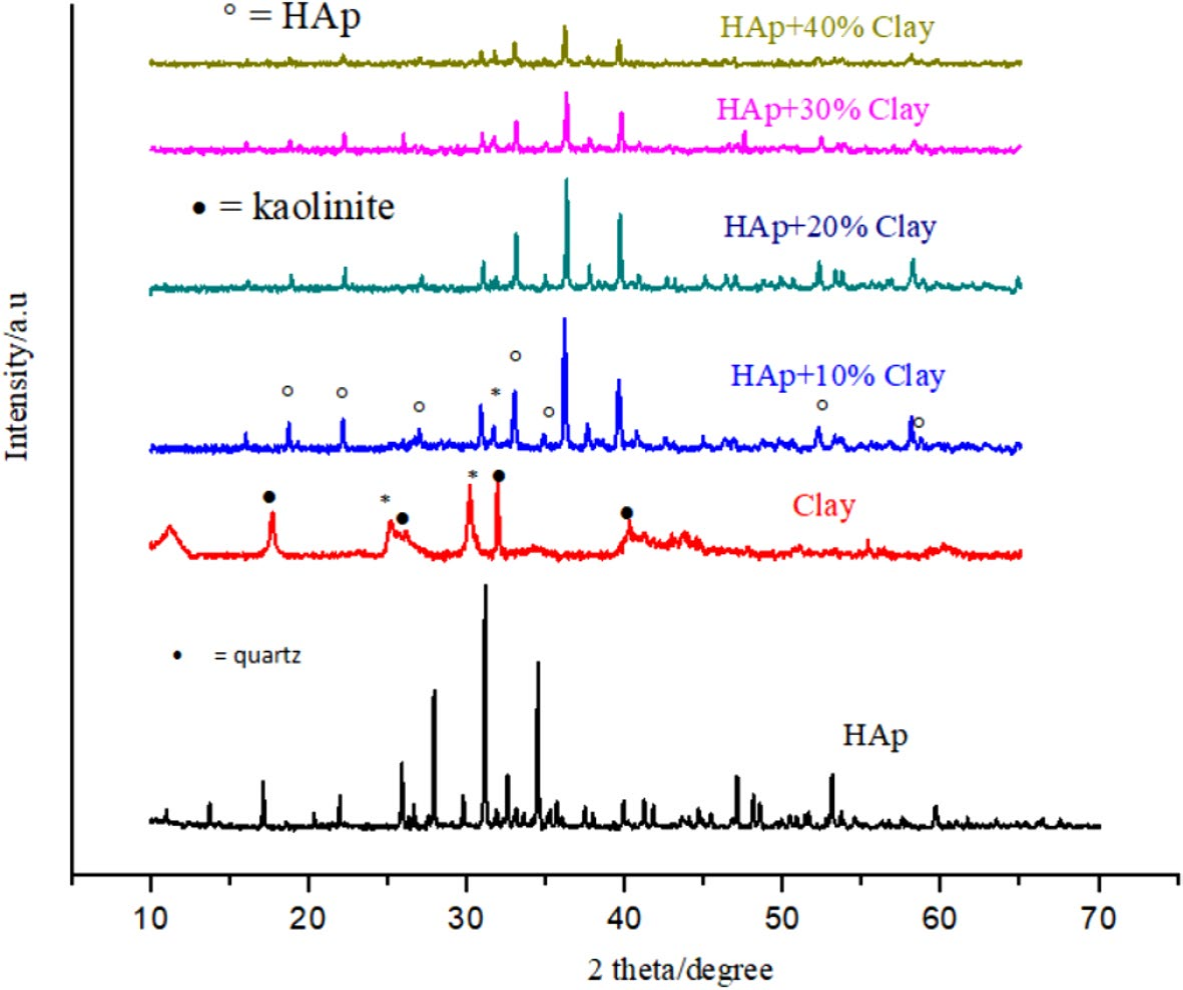
XRD of natural clay, synthesized HAp and HAp/clay nanocomposites.

Identification of crystalline phases of the prepared HAp-Clay composite material, pure HAp and raw clay were done using X-ray diffraction as presented in Fig. 3 which clearly reveals typical characteristic diffraction patterns of calcium hydroxyapatite, kaolinite clay and nano crystalline hydroxyapatite/clay composite. Prominent peaks at 2θ = 28, 31, and 33° on diffractogram are characteristic of pure HAp^20^. The presence of other less intense peaks corresponding to HAp confirms the presence of calcium hydroxyapatite (JCPDS file no. 09-432). The appearance of many sharp peaks is an indication of well crystalline apatite after heat treatment^20^. From the XRD analysis of the natural sample, the diffraction peaks at 2θ = 12.02°, 19.03°, 26.87°, 30.01° and 40.01° clearly shows the existence kaolinite clay^22^. Peaks appearing at 2θ = 26.20°, 2θ = 28.01° and 31.01° are characteristics of quartz compounds^23,26^. The XRD diffraction pattern of HAp/clay nanocomposite is equally presented in Fig. 3. The result showed a complete disappearance pattern from the starting materials of HAp and kaolinite peaks thus suggesting the formation of HAp/clay nanocomposite, although, peaks corresponding to the precursor materials were observed. It was observed that as the amount of clay incorporated into the apatite structure raises, the more shrinking the structure becomes. To further investigate the impact of clay incorporation into the apatite structure, the crystallite size was computed using the Scherrer’s equation from the XRD data, $D = k\lambda /\cos \theta$, such that K is a constant, λ is the X-ray wavelength, D is represents the full-width half maximum and θ denotes the Bragg’s angle. The values obtained are as listed in Table 1 which clearly shows that crystallite sizes decrease as the amount of clay sample incorporated into the apatite increases. The lattice defects resulting from the reaction between the HAp and clay sample was evaluated further by estimating the unit cell parameters (a, c) and the unit cell volumes (V) as depicted in Table 1. On comparing the lattice parameters values with standard JCPDS no. 09-432 for pure HAp, it was observed that these values were close. It was noticed that while parameter ‘a’ increases with increase in clay amount incorporated into the apatite lattice, parameter ‘c’ which is the unit cell volume on the contrary showed a drastic reduction.

**Table 1:** Lattice properties of HAp and HAp/clay nanocomposites.

Samples	FWHM (°)	Parameter			Crystallite size (nm)
		a (nm)	c (nm)	V (nm)	
HAp (JCPDS no. 09-432)	–	0.9418	0.6884	–	–
HAp	0.116	0.9416	0.6882	1.569	82
HAp + 10% Clay	0.1014	0.9427	0.6873	1.570	79
HAp + 20% Clay	0.1021	0.9424	0.6871	1.572	76
HAp + 30% Clay	0.1024	0.9433	0.6868	1.577	72
HAp + 40% Clay	0.1032	0.9435	0.6862	1.580	69

### Scanning electron microscopy

**Figure 4:**
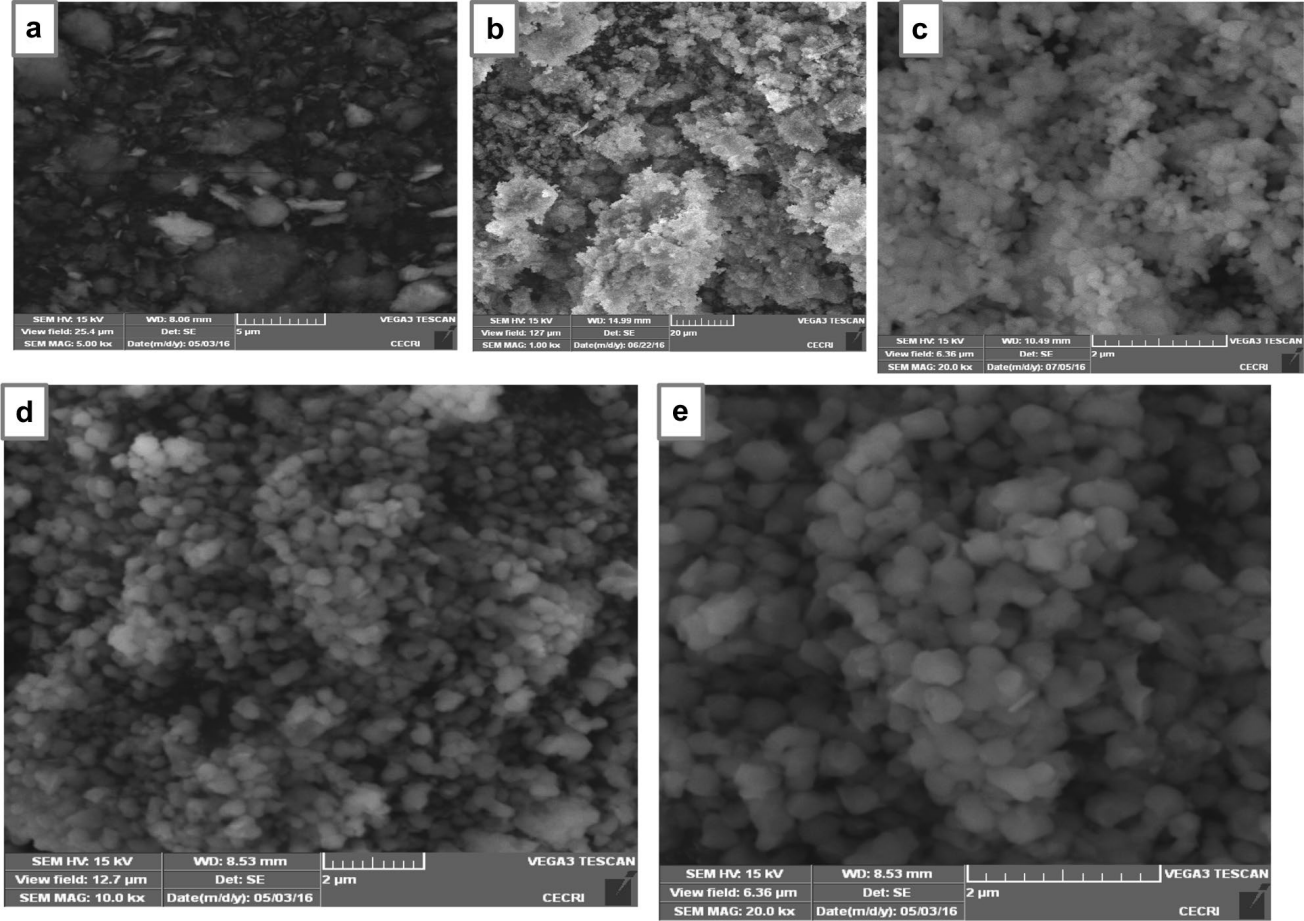
SEM images of (**a**) natural clay, (**b**) synthesized 10% clay + HAp, (**c**) 20% clay + HAp and (**d**) 30% clay + HAp nanocomposites.

**Figure 5:**
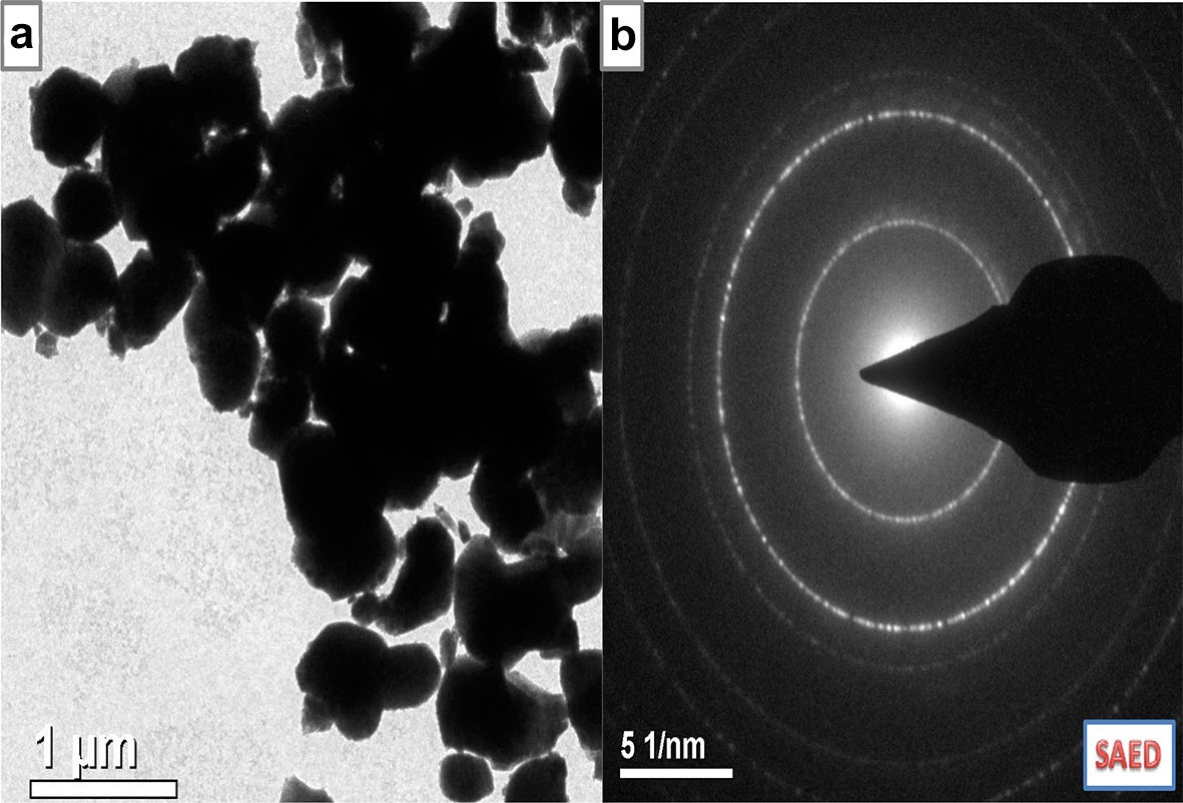
(**a**) TEM and (**b**) SAED images of synthesized 30% clay + HAp nanocomposites.

The SEM micrographs of the clay, prepared HAp and HAp/clay composite at different ratios are as depicted in Fig. 4. The morphological structure of the clay indicates particles having irregular platelets shapes and flakes (Fig. 4a). The secondary electron micrograph of the as-prepared apatite indicates that the well crystallized HAp particle contains flower-like morphology (Fig. 4b). However, after the incorporation of clay sample in the prepared HAp mineral, round shape particles were formed with particle becoming bigger on increasing the quantity of clay in the apatite (Fig. 4c, d). This clearly shows the formation of a composite and also supports the XRD result which shows increase in the lattice parameter ‘a’ as the quantity of clay increases. To further provide insight into the morphology of the composite, TEM analysis was carried out as shown in Fig. 5a with its corresponding selected area electron diffraction (SAED) pattern represented in Fig. 5b. The TEM investigation confirms the SEM results that the morphology of the as-prepared apatite composed of agglomerated round particles with crystal length in the range of 10–45 nm and width between 6 and 34 nm.

### Chemical composition

**Figure 6:**
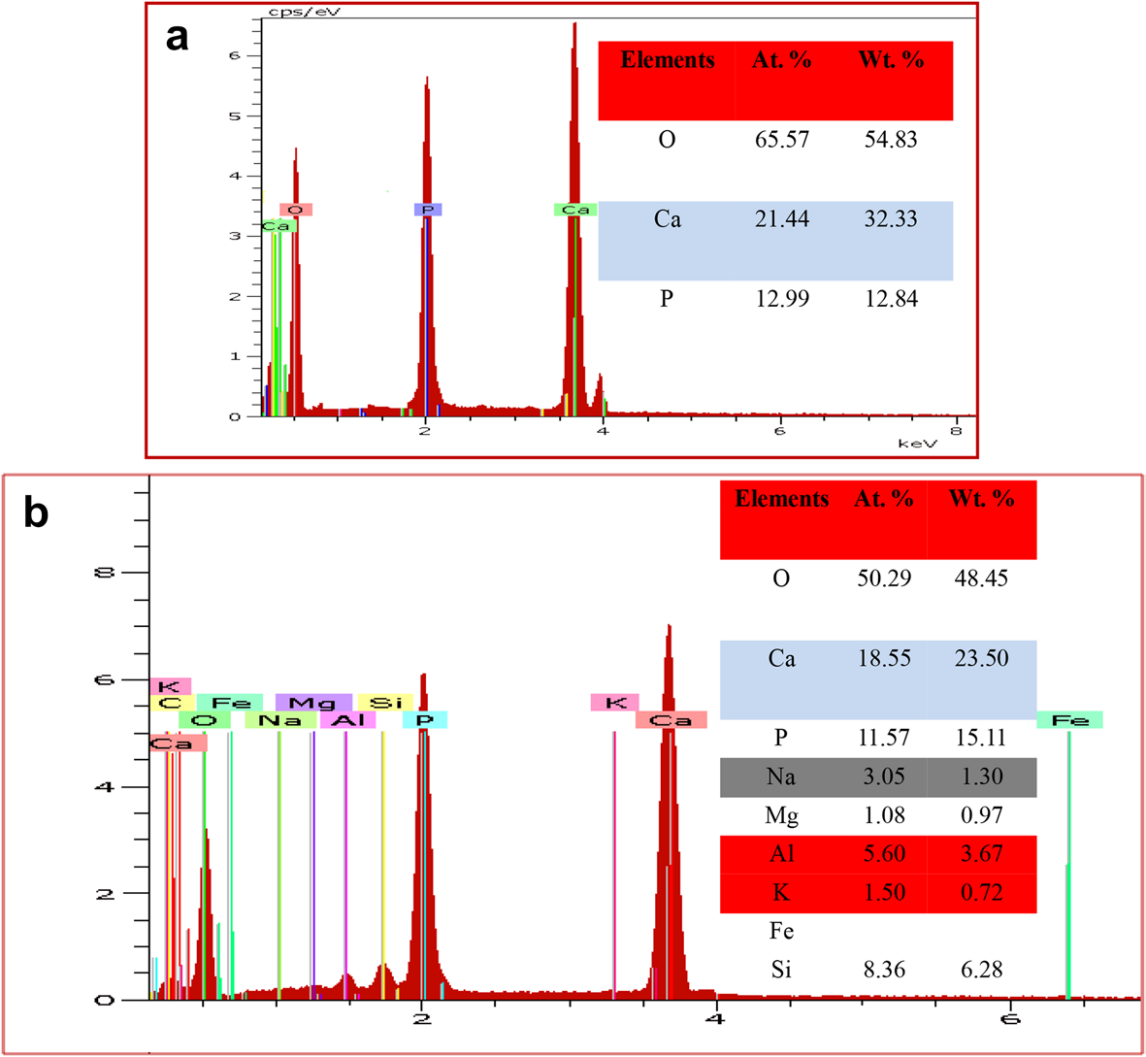
EDX of (**a**) synthesized HAp and (**b**) synthesized 30% clay + HAp nanocomposites.

Table 2 presents the X-Ray Fluorescent (XRF) analysis of the chemical composition of raw clay sample. It could be seen that the clay sample gave SiO_2_ (52.04%) and Al_2_O_3_ (22.18%) as the major oxides present. Other oxides detected by the XRF analysis are Fe_2_O_3_, Na_2_O, K_2_O and MgO respectively. The presence of all these oxides confirms the dominant of kaolinite structure as depicted in XRD analysis. Figure 6a shows the EDX chemical composition of pure hydroxyapatite synthesized, while the chemical composition of the HAp/clay composite produced is represented in Fig. 6b respectively. The EDX of the pure HAp revealed the presence of O (65.57%), Ca (21.44%) and P (12.99%) as the elements present in the pure apatite with a Ca/P of 1.65. However, the EDX of the composite shows the presence of other elements from the precursor material of clay such as K, Na, Ca, Mg, Si and Al. The ratio of calcium to phosphorous in the as-prepared composite is 1.60 indicating a deficient calcium phosphate due to the presence of the other metals in the structure. This further confirms the results from XRD and FT-IR analyses. It is worthy to note that the presence of these mineral elements in the composite could enhance the biological activities of the biomolecule.

**Table 2:** XRF Analysis of raw clay.

Chemical formula	Chemical composition (%)
Al_2_O_3_	22.18
SiO_2_	52.04
Fe_2_O_3_	10.48
Na_2_O	8.01
K_2_O	4.81
MgO	2.47

### Mechanical properties

**Figure 7:**
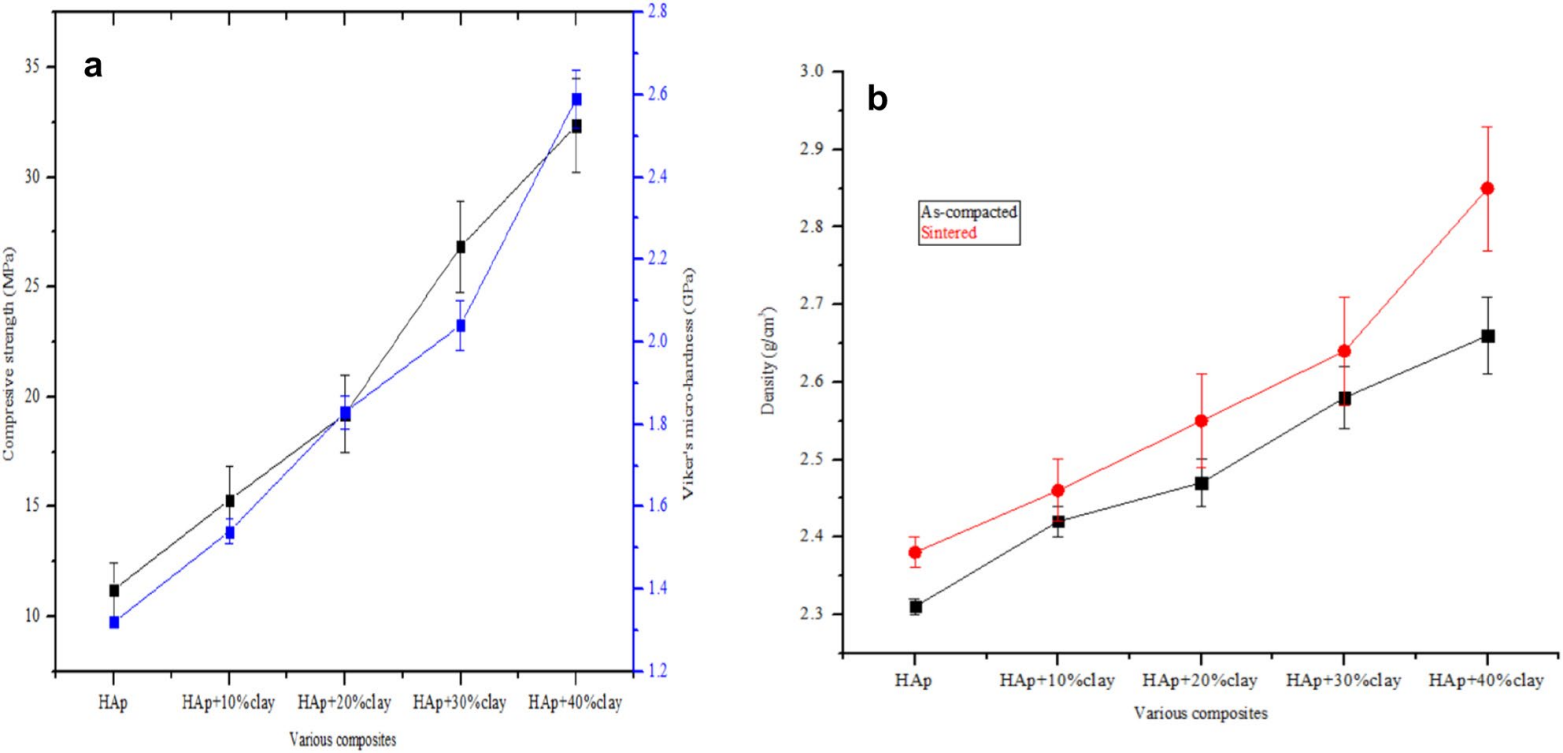
Plots of (**a**) mechanical properties and (**b**) densities of synthesized HAp and sintered HAp/clay nanocomposites.

The mechanical properties of the fabricated composite were studied by evaluating the compressive strength and Vicker’s micro-hardness properties of the composite as shown in Fig. 7a. It was observed that the compressive strength increased drastically from HAp + 10%clay (15.32 MPa) to HAp + 40% clay (32.35 MPa). However, when clay was absent, a compressive strength of11.24 MPa was achieved for pure HAp. Similarly, the Vickers micro-hardness computed rose from 1.54 GPa for HAp + 10%clay to 2.59 GPa for HAp + 10%clay respectively. In this study, considerable improvements in the mechanical properties were noticed after the incorporation of 30 and 40% of clay into the HAp structure. Even though the mechanical strength of the fabricated composite improved with the addition of clay samples, a point will be reach when further increase in the amount of clay will lead to the formation of poor the deformation of the structure of the apatite. The density of the as-compacted and heat treated HAp-C composite is shown in Fig. 7b. It was observed that the density of the as-compacted HAp-C composites increased from 2.31 to 2.66 g/cm^3^, while that of the sintered composites increased from 2.38 to 2.85 g/cm^3^ with the heat-treated nanocomposite demonstrating higher densities. Furthermore, it was noticed that the densities of the clay-substituted apatite rose with an increase in the percentage of clay incorporated into the apatite structure thus resulting into some structural deformations^20^.

**Figure 8:**
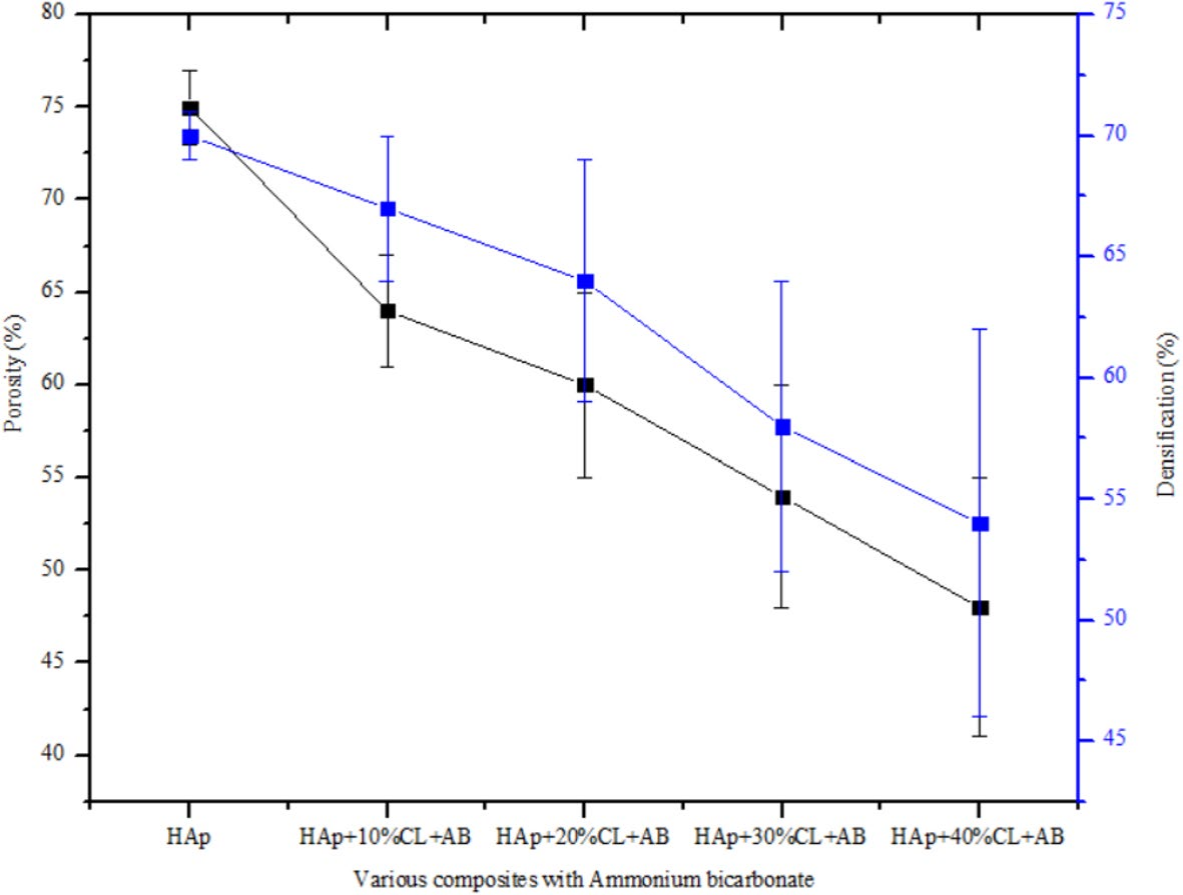
Plots of porosity and densification of the synthesized HAp and sintered HAp/clay nanocomposites.

**Figure 9:**
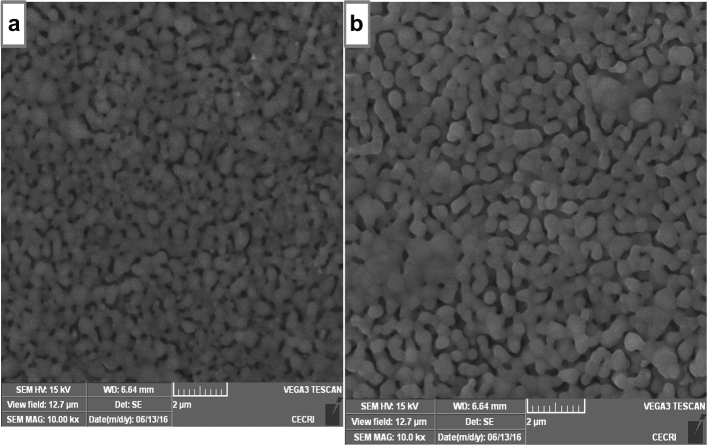
SEM images of the surface of HAp/clay nanocomposite mixed with ammonium bicarbonate.

In other to improve the porosity of the fabricated apatite, samples were reacted with 30% ammonium bicarbonate as shown in Fig. 8. It was observed that the presence of ammonium bicarbonate creates some pores on the surface of the nanocomposite which resulted in the formation of an interconnected porous scaffold structure. Although, a porosity of 75% was obtained in case of the pure HAp, however this was considered to be fragile when subjected to compressive strength testing. Upon the addition of clay and ammonium bicarbonate to the apatite, the porosity decreases from 64 to 48% with improved mechanical property. In other to ensure better mechanical property of the composite, 30% clay with ammonium bicarbonate was selected which gave a porosity of 54% and densification of 58% respectively. The SEM image of the surface of HAp-C with ammonium bicarbonate is shown in Fig. 9. The morphology showed an interconnected porous structure which could enhance the growth of cells, vascularization of diffusion of metabolic products which are essential during bone implants ^20,27^.

### Biocompatibility assay

**Figure 10:**
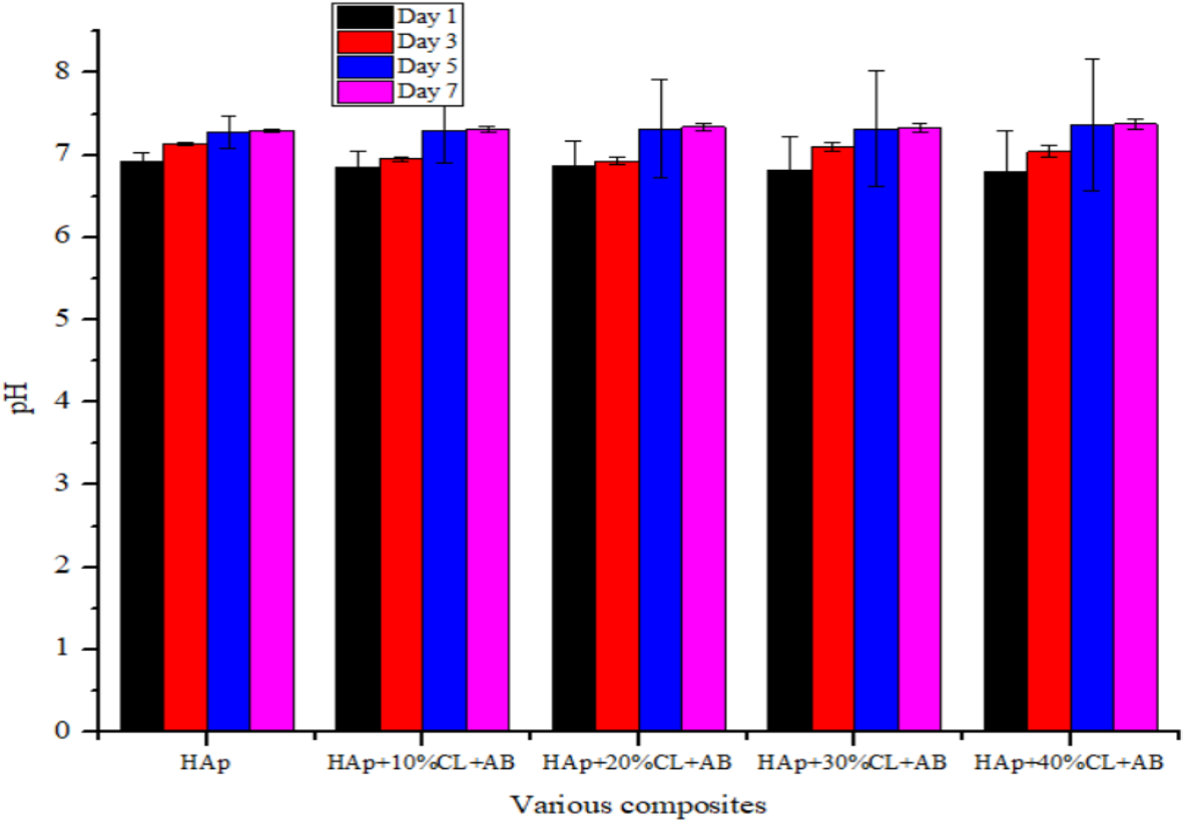
pH of HAp/clay nanocomposite mixed with ammonium bicarbonate soaked in SBF media.

The physiological behaviour of the fabricated apatite composites were investigated by monitoring the changes in the pH values of the composites using saline solution for 7 days as shown in Fig. 10. After day 1 in saline solution, the values of the pH of HAp, HAp + 10%clay + AB, HAp + 20%clay + AB, HAp + 30%clay + AB and HAp + 40%clay + AB composites were found to be 6.93, 6.85, 6.87, 6.82 and 6.80 respectively. There was steady increase in the values of the saline pH of the various biomolecules until day 5 and were observed to be fairly constant at day 7 with pH range of 7.30–7.38.

**Figure 11:**
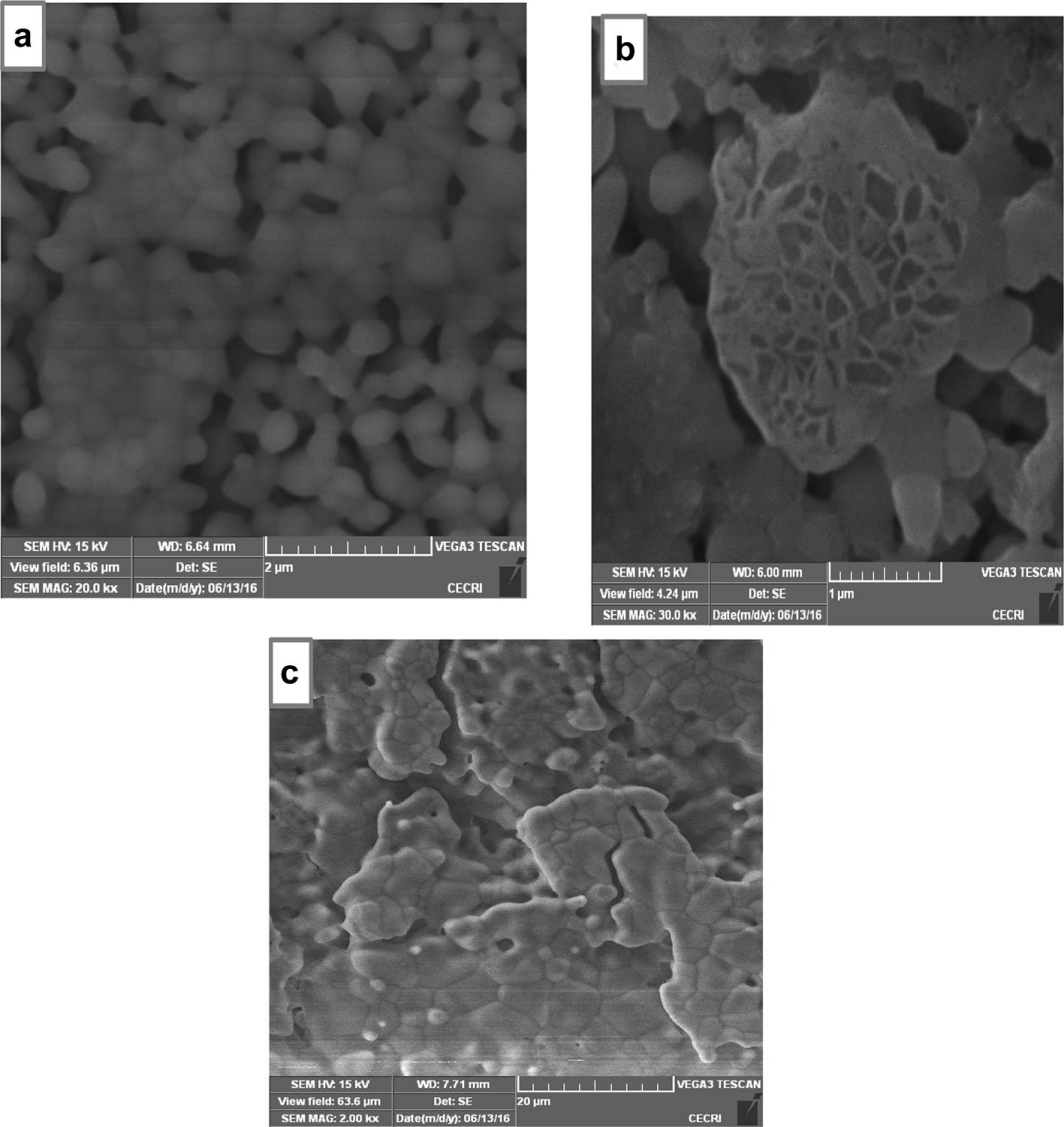
SEM images of the surface of HAp/clay nanocomposite mixed with ammonium bicarbonate after soaking in SBF solution for (**a**) 3 days, (**b**) 5 days and (**c**) 7 days.

**Figure 12:**
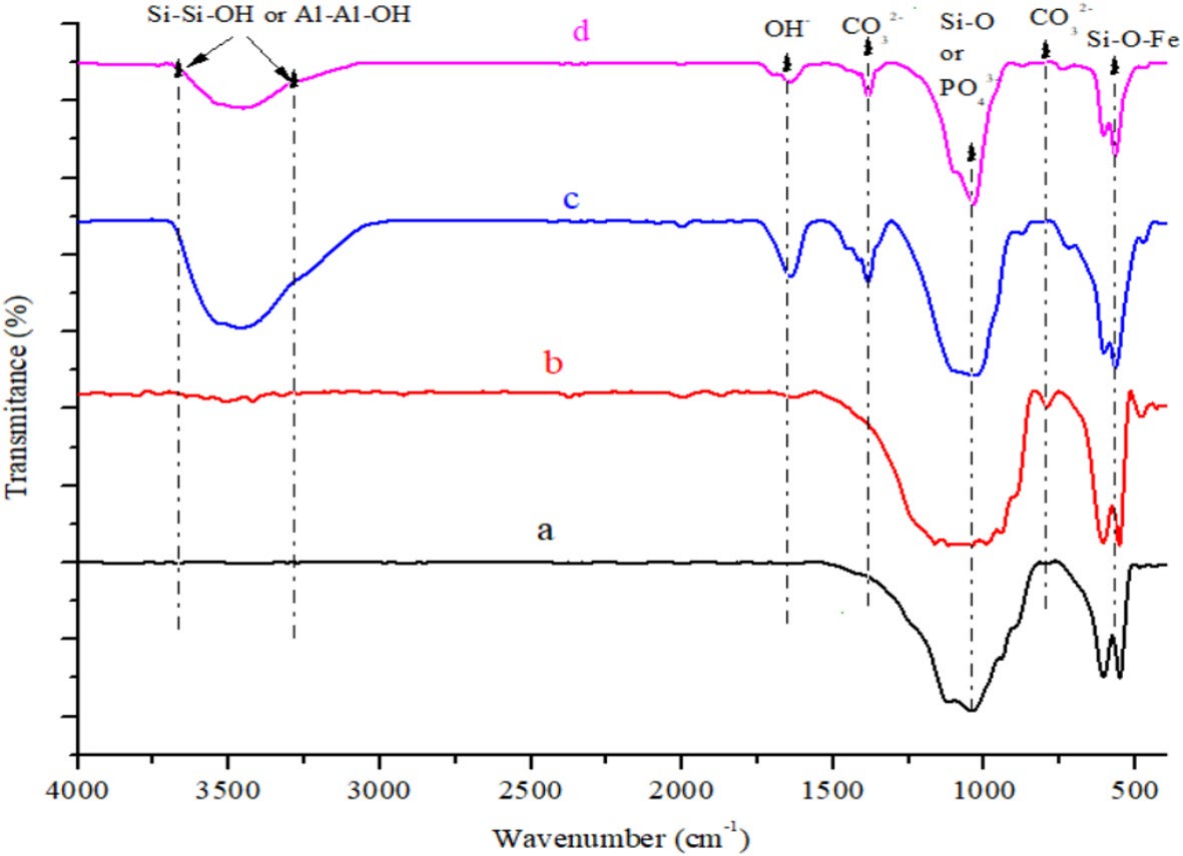
FT-IR spectra of (**a**) HAp + 30% clay + AB before soaking, (**b**) HAp + 40% clay + AB before soaking, (**c**) HAp + 30% clay + AB after soaking and (**d**) HAp + 40% clay + AB after soaking for 7 days.

**Figure 13:**
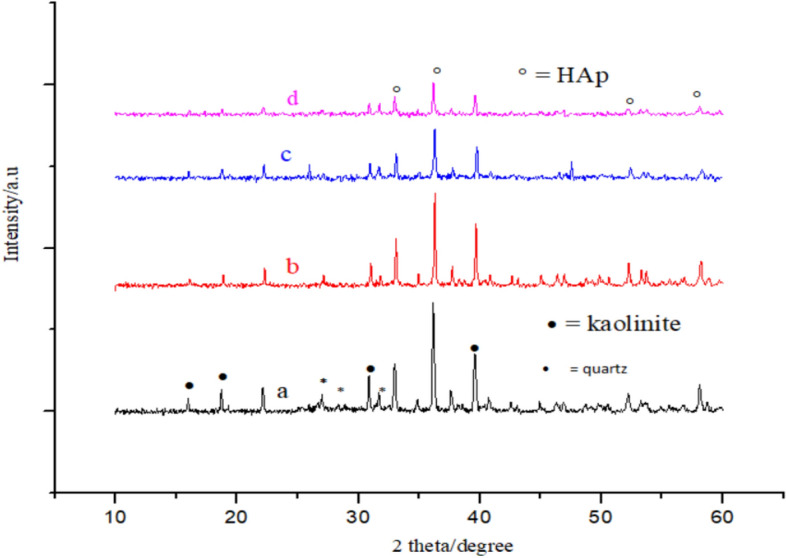
XRD of (**a**) HAp + 30% clay + AB before soaking, (**b**) HAp + 40% clay + AB before soaking, (**c**) HAp + 30% clay + AB after soaking and (**d**) HAp + 40% clay + AB after soaking for 7 days.

Though the saline solution was acidic at the beginning of the reaction process due to the dissolved carbon dioxide, the pH of the saline solution containing the nanocomposites gradually became neutral and fairly alkaline over time as a result of the presence of Lewis basis structures in HAp-C composites which helps in neutralizing the acidic solution^20,28^. It is imperative to note that the final values of the pH of HAp + 10%clay + AB, HAp + 20%clay + AB, HAp + 30%clay + AB and HAp + 40%clay + AB were close to the pH of human plasma^28,29^. According to Arundhati Bhowmick et al.^28^ composites having low alkaline pH, should not produce any toxicity to human body during bone implant. Morphological analyses of the surface of the fabricated nanocomposites after soaking in SBF solution for 3-, 5- and 7-days using SEM is shown in Fig. 11a–c which revealed perfect interactions between the nano-composites fabricated and the SBF solution. Agglomerations of particles were seen after 3 days on the surface of porous scaffold which gradually filled up the entire pore surface after 7 days. The presence of functional groups on the surface of the composites such as PO_4_^3−^, –OH and –Si–O might have interacted with cations of the SBF solution resulting in the exchange of ions. This behaviour in the prepared scaffolds were cross-examined using FT-IR of the SBF soaked HAp/clay/ammonium bicarbonate composites as represented in Fig. 12. In the FT-IR spectra, broad band centered between 3365 to 3645 cm^−1^ was assigned to O–H stretching frequency of apatites deposits from the SBF solution on the surface of nanocomposites. This O–H stretching frequency was initially absent on the surface of the pure composites. The peaks observed at 1455 cm^−1^, 1448 cm^−1^ and 878 cm^−1^ suggests the presence of the B-type carbonate^29^. Si–O stretching of the clay was noticed around 1055 cm^−1^ which appeared overlapped with the PO_4_^3−^ stretching of the HAp. This further confirms the interaction of the as-prepared composites with SBF media. Absorption bands corresponding to interlayer water of clay as well as adsorbed water by the nanocomposites were observed at 1621 cm^−1^ and these FT-IR peaks were absence in the pure nanocomposites prepared. Generally, the treatment of the HAp-C scaffolds with SBF media resulted in shift of FT-IR peak positions most especially at the phosphate, hydroxyl and carbonate groups. The observed carbonate bands after SBF treatment is an indication that the crystal particles deposited on the surface of the apatite during immersion period are carbonated apatites which resemble those of natural apatite^20^. The XRD patterns of HAp-C composites after soaking in SBF media is illustrated in Fig. 13. The characteristic diffraction peak of HAp and its composites were observed respectively. For HAp-C composites after SBF treatment, diffraction peaks appeared to be weaker and fewer with low intensity than pure HAp-C composites which might be due to high dispersion of HAp-C composites in the SBF media.

Therefore, the investigation of HAp-C composites in the SBF media by SEM, FT-IR and XRD confirm the bioactivity of the as prepared scaffolds. According to Pradnya et al., 2010, surface chemistry plays vital role during in the process of apatite incubation in SBF media and that bone bonding property is a function of the functional groups available on the surface of the material. Chemical reactions such as nucleation and spontaneous precipitation take place during the process of apatite incubation in SBF media^20,30^.

## Conclusions

Biomaterials of HAp, HAp-C and HAp-C-AB nanocomposites were developed to derive a better biological and mechanical property materials that could be deploy in bone tissue engineering application. The structural compositions of the fabricated nanocomposites were done using FT-IR, XRD, SEM, and TEM. The FT-IR spectra of HAp/clay composites show functional groups of HAp and clay such as PO_4_^3−^, –OH, Si–Si–OH, Al–Al–OH, Si–O and Si–O–Al. The XRD results showed a complete different pattern from the starting materials of HAp and kaolinite peaks thus suggesting the formation of HAp/clay nanocomposite. SEM and TEM images revealed that the nanocomposites so formed are made up of round shape particles which grow bigger on increasing the quantity of clay in the apatite structure. The mechanical properties of the as-prepared HAp-C nanocomposites were enhanced when compared with pure HAp after the addition of clay into the apatite structure. In addition, proliferation of apatites particles onto the surface of the nanocomposites was observed after immersion in SBF media for 7 days. Thus, HAp-C and HAp-C-AB nanocomposites could be regarded as potential biomaterials in bone tissue engineering.

## Data Availability

The datasets used and/or analyzed during the current study available from the corresponding author on reasonable request” in the data availability section.
